# Interpretable Drug–Target Prediction via Geometric Alignment: A Linear Alternative to Deep Architectural Complexity

**DOI:** 10.1002/minf.70060

**Published:** 2026-10-29

**Authors:** Aashutosh Ghimire, Chris Alvin, Adam Bess, Supratik Mukhopadhyay

**Affiliations:** ^1^ Department of Biological Sciences Louisiana State University Baton Rouge Louisiana USA; ^2^ Department of Computer Science Furman University Greenville South Carolina USA; ^3^ Department of Computer Science Louisiana State University Baton Rouge Louisiana USA; ^4^ Department of Environmental Sciences Louisiana State University Baton Rouge Louisiana USA

**Keywords:** computational drug discovery, contrastive learning, drug‐target interaction, foundation models, interpretable machine learning, kinase inhibitor selectivity, latent space alignment

## Abstract

Designing selective kinase inhibitors is a multiobjective optimization problem constrained by the structural conservation of the human kinome, along with the lack of high‐quality interaction data. Traditional deep learning methods often train complex neural networks directly on small datasets and fail to generalize to new chemical scaffolds. To this end, we present *Geometric Manifold Alignment*, a novel linear approach that shifts away from architectural complexity. We introduce *Multi‐View Linear Manifold* (*MVLM*), which aligns the latent spaces of ChemBERTa (small molecules) and ESM‐2 (protein sequences) into a shared geometric interaction space. We pretrain MVLM on the *Universal Foundation Set* of 473 923 high‐confidence interactions, where binding affinity is represented as geometric proximity between drug and target embeddings. Achieving an AUROC of 0.936 on KIBA and 0.928 on Davis, MVLM establishes the new state‐of‐the‐art on industry‐standard benchmarks, outperforming existing methods (*p* < 0.001). In this work, interpretability is geometric where interaction scores are cosine similarities in a shared manifold, and the learned structure recovers kinase phylogenetic relationships without supervision. We also demonstrate the model’s utility as a transparent discovery engine by retrieving known clinical targets of FDA‐approved drugs. Our findings suggest that aligning pretrained chemical and biological representations provides a scalable and interpretable approach to advancing computational drug discovery.

## Graphical abstract

The Multi‐View Linear Manifold (MVLM) aligns frozen chemical and protein language model embeddings in a shared geometric space using two linear projections. Binding is scored by a single dot product. MVLM achieves state‐of‐the‐art drug–target interaction prediction on KIBA, performs competitively on Davis, and recovers kinase phylogenetic structure without supervision.

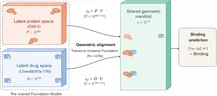

## 1 Introduction

The human kinome contains 518 protein kinases [1] that serve as central regulators of cellular signaling. When unregulated, these kinases fuel disease progression, particularly in cancer [2]. Kinase inhibitors have become a cornerstone of modern oncology since the approval of imatinib in 2001 [3]. However, designing selective inhibitors remains a fundamental challenge: most kinases share highly conserved active sites [4], and it is extremely difficult to develop a molecule that binds to a specific target without toxicity at off‐target sites. The combinatorial scale of possible drug–kinase interactions makes an exhaustive analysis intractable [5], thereby necessitating efficient computational approaches to drug–target interaction (DTI) prediction.

Pioneering models like DeepDTA [6] and GraphDTA [7] approached DTI prediction by training deep neural networks without pretrained weights. Although promising, these models were frequently trained on small datasets (less than 100 000 interactions) [8], thus limiting their ability to generalize to novel chemical scaffolds [9]. Consequently, these models lack the generalizability and interpretability (due to the complexity of the neural networks trained) needed for practical drug discovery.

We propose *Geometric Manifold Alignment* via the *Multi‐View Linear Manifold* (MVLM) framework. Rather than training a neural network from scratch, MVLM uses pretrained Foundation Models [10] (ChemBERTa [11] for small molecules and ESM‐2 [12] for protein sequences) and aligns their latent spaces through linear projections into a shared 512‐dimensional geometric space, where binding affinity corresponds to geometric proximity. Pretrained on a Universal Foundation Set of 473 923 high‐confidence interactions, MVLM achieves state‐of‐the‐art performance on the KIBA and Davis benchmarks while providing interpretability via the learned geometric structure of the human kinome.

The novelty of the MVLM framework lies in showing that, for kinase‐focused drug–target prediction, one can achieve strong performance without involving task‐specific deep interaction architectures by instead aligning pretrained chemical and protein embeddings in a shared linear manifold. MVLM fuses multiview drug encoding, large‐scale universal pretraining on curated kinase interactions, and a hybrid contrastive‐discriminative loss to learn the representation of a geometrically structured interaction space. This approach provides a conceptually different and computationally simpler alternative to existing deep DTI pipelines, while at the same time enabling analysis of the learned manifold structure. The data and models from our work is publicly available at https://github.com/deepdrugai/MVLM‐DT.

## 2 Methods

### 2.1 Building the Universal Foundation Set

Our training set was constructed using BindingDB [13] (January 2026 release) targeting 447 of the 518 human kinases with sufficient high‐confidence interactions in the database. Public databases frequently contain conflicting measurements for the same drug–target pair. To resolve this, we formulated a *priority coalescing strategy* ranking measurement by affinity type:

(1) $$K_{i}>K_{d}>{\text{IC}}_{50}>{\text{EC}}_{50}$$

When multiple measurements existed for a given pair, the highest‐ranked value was retained. Our coalescing strategy yielded 473 923 high‐confidence interactions. All measurements were normalized to a pAffinity score; *K* denotes the measured affinity in nM:

(2) $$\text{pAffinity}=9-\log_{10}(\text{K})$$

An active binding threshold of 100 nM (pAffinity≥7.0) was used to define positive interactions.

**Train/Test Split and Leakage Control.** To prevent data leakage, we used a cold‐drug splitting protocol by partitioning the Universal Foundation Set into a 90% training set and 10% testing set based on unique SMILES representations (achieved using canonicalization with RDKit), ensuring no molecule appeared in both splits. Leakage analysis confirmed no overlap between training and testing compounds, ensuring that the testing data is unseen during training.

**Problem Formulation: Binary DTI Classification Over DTA Regression.** DTI is cast as a binary classification task rather than continuous drug–target affinity (DTA) [14, 15] for two reasons. First, the Universal Foundation Set combines measurements from multiple experimental assay types (*K*_*i*_, *K*_d_, and IC_50_). These measurements are not thermodynamically equal; therefore, attempting to predict the precise numerical values of such mixed measures would create significant noise. Second, our geometric manifold approach naturally maps active drug–target pairs to neighboring regions, while inactive pairs are mapped to distant regions in the shared space. This makes interaction prediction an inherently binary geometric objective. Ranking candidate targets thus follows directly from geometric similarity scores.

### 2.2 Input: Foundation Model Encoders

Two pretrained foundation models serve as input encoders. For drug representation, we use ChemBERTa‐77 M [11], a transformer model trained on 77 million chemical compounds. We then form drug embeddings using a multiview approach by concatenating the global molecular token ([CLS]) with the mean of all atom‐level tokens. This results in a 768‐dimensional vector capturing both global and local structural and chemical features. The single‐view variant, used as an ablation baseline, retains only the [CLS] token as the drug representation. For protein representation, we use ESM‐2 (35M), a protein language model that is pretrained on evolutionary sequence data that generates a 480‐dimensional vector for each kinase from its amino acid sequence. Note that our multiview approach applies to the drug encoder, where it captures both global molecular and local atomic features. ESM‐2 produces a single fixed‐length embedding per protein sequence and does not require a multiview representation.

### 2.3 The Linear Manifold Architecture

MVLM applies a linear manifold alignment layer over pretrained deep chemical and protein language model embeddings (ChemBERTa‐77 M and ESM‐2), keeping the foundation encoders frozen. The learned interaction is therefore linear with respect to the embedding space, while the underlying representations are extracted by deep transformer architectures. As shown in Figure 1, MVLM learns two projection matrices, $U\in R^{768\times 512}$ and $V\in R^{480\times 512}$, that respectively map drug and protein embeddings into a shared geometric space. In particular, a drug embedding $x_{d}\in R^{768}$ is projected as *z*_*d*_ =* x*_d_ *U* while a protein embedding $x_{p}\in R^{480}$ is projected as *z*_p _=* x*_p_ *V*. Each projection results in a 512‐dimensional representation in the shared geometric space. Prior to computing affinity scores, both representations are L2‐normalized (${\hat{z}}_{d}=\frac{z_{d}}{\left\|z_{d}\right\|}$, ${\hat{z}}_{p}=\frac{z_{p}}{\left\|z_{p}\right\|}$) resulting in a final projection onto the unit hypersphere in $R^{512}$. We note that on this hypersphere, the dot product reduces to cosine similarity, and thus, the dot product is a geometrically meaningful measure of alignment between vectors and reflects the angle between drug and protein representations in the shared manifold. Binding affinity is then computed between a drug–target pair as:

(3) $$\text{Affinity}=\frac{\left\langle {\hat{z}}_{d},{\hat{z}}_{p}\right\rangle }{\tau }$$

**FIGURE 1:**
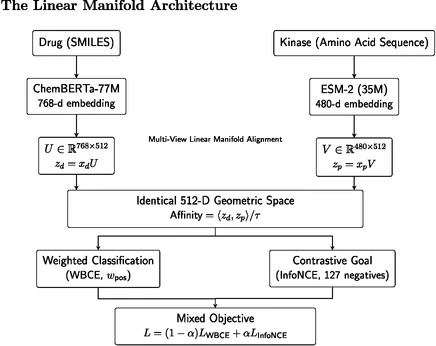
Overview of multiview linear manifold model. Pretrained protein embeddings of 480 dimensions from ESM‐2 and drug embeddings of 768 dimensions from ChemBERTa‐77 M are projected into a shared 512‐dimensional interaction space using learnable linear transformations producing aligned representations of the protein and drug in the same space. Binding affinity is defined by the scaled dot‐product similarity between the projected drug and protein representations, where the temperature parameter controls scaling. In training, each positive drug–kinase interaction is compared to 127 randomly chosen kinases that do not have known interactions with the same drug for better separation of true binding pairs from noninteracting pairs within the shared geometric manifold.

with τ being a learned temperature parameter that scales the magnitude of affinity scores. Thus, active pairs are geometrically characterized by small angles (high cosine similarity) and inactive pairs by larger angles.

The model is trained to satisfy the following hard constraint:

(4) $$\langle {\hat{z}}_{d},{\hat{z}}_{p}\rangle \approx 1\;\text{⇔}\;\text{Active Interaction}$$

Geometric proximity in the shared manifold thus directly reflects biological binding affinity.

**Optimization Strategy.** The analytically optimal solution for aligning the Drug Space (*X*) and Protein Space (*Y*) is the Moore–Penrose pseudoinverse solution ($W^{*}=X^{†}Y$). However, computing this solution directly requires inverting a matrix of size N×N. At the scale of the Universal Foundation Set ($N>4.7\times 10^{5}$), this becomes computationally infeasible.

To bypass this, we approximate the analytically optimal alignment using Stochastic Gradient Descent (SGD). By decomposing the alignment into two learnable low‐rank projections (*U* and *V*) and optimizing them in mini‐batches, the computational cost is reduced to *O*(*N*) per epoch. This enables the model to converge to a stable manifold alignment that closely approximates the analytically optimal solution while remaining scalable to datasets containing millions of interactions.

### 2.4 Hybrid Geometric Loss

Model training employs a hybrid loss function that balances classification accuracy and geometric structure.

(5) $$L=(1-\alpha )\cdot L_{\text{WBCE}}+\alpha \cdot L_{\text{InfoNCE}}$$

where *α* = 0.3 controls the tradeoff between our two objectives.

**Weighted Binary Cross‐Entropy (WBCE).** Since active drug–target pairs are rare, a positive class weight *w*_pos_ = 1.5 is applied to prevent the model from defaulting to predicting all interactions as inactive, ensuring sensitivity to true binding events.

**InfoNCE (Contrastive Learning).** Each active drug–target pair is compared against 127 randomly sampled negative kinases [16]. This forces the model to map the true target to a neighboring region while also pushing inactive targets to remote regions of the shared manifold. This enforces geometric selectivity in the learned space.

### 2.5 Training Protocol

We adopt a two‐step transfer learning approach. In the first step, **Universal Pre‐training**, the model is trained on the full Universal Foundation Set of 473 923 interactions drawn from BindingDB [13] to learn the general rules of kinase–ligand binding. In the second step, we use **Benchmark Fine‐tuning**, with the pretrained model being independently fine‐tuned on the Davis [17] or KIBA [18] benchmarks using a reduced learning rate (1 × 10^−5^). This allows adapting the model to distribution‐specific benchmarks while preserving the generalizable binding geometry learned during pretraining. Each model was implemented using PyTorch and trained on NVIDIA V100 (16 GB) GPUs with AdamW (learning rate =1 × 10^−3^, weight decay =1 × 10^−5^), batch size =256. The ReduceLROnPlateau callback reduced the learning rate by half if the validation AUROC did not improve after 5 epochs. Training was limited to 100 epochs with a patience set at 15 epochs. The activity threshold follows established practices: ≤100 nM for Davis [17] and KIBA score ≤12.1 for KIBA [18]. The value of *w*_pos_ = 1.5 was determined using a holdout validation fold; values less than 1.0 affected precision negatively, whereas values above 2.0 led to overestimation of positives. The loss balance *α* = 0.3 provided an appropriate bias toward classification loss while retaining geometric regularization. The number of in‐batch negatives for InfoNCE is set to 127, the maximum possible given a batch size of 128. We note that the absence of a BindingDB record does not imply inactivity, as many kinase–inhibitor pairs remain untested rather than verified inactive. Following [19], we treat unlabeled pairs as negatives under the closed‐world assumption, consistent with prior contrastive DTI methods.

### 2.6 Declaration of AI‐Generated Content

During preparation of this manuscript, the authors used Claude Opus 4.8 solely to improve the language, grammar, and readability of the author‐written text. The authors reviewed and edited all content and take full responsibility for the manuscript.

## 3 Results

### 3.1 Performance on the Universal Kinome (Cold‐Drug Split)

Prior to evaluation on external benchmarks, we assessed the ability of MVLM to generalize across the 447 human kinases represented in the Universal Foundation Set. We used the 10% cold‐drug holdout, containing approximately 47 000 interactions, as a rigorous unseen evaluation set. This holdout is strictly partitioned by RDKit‐canonicalized SMILES, ensuring no drug molecule observed during training appears in the test set. When the split was further constrained to exclude both drugs and proteins from training, the performance decreased by approximately 0.12 (from 0.919 to 0.799; Table 1). This decline is consistent with the increased difficulty of complete cold generalization, where the model must simultaneously extrapolate across unseen chemical scaffolds and unseen protein targets.

**TABLE 1:** Generalization performance of MVLM across four data partitioning regimes on the Universal Foundation Set. Results are mean ± SD across three random seeds.

Split regime	**AUROC** **↑**	**AUPRC** **↑**
Cold‐drug/warm‐target	0.9189 ± 0.0012	0.9746 ± 0.0008
Scaffold split	0.8082 ± 0.0009	0.9339 ± 0.0010
Cold‐target/warm‐drug	0.8437 ± 0.0024	0.9229 ± 0.0010
Double‐cold split	0.7986 ± 0.0014	0.9273 ± 0.0008

We tested the MVLM model on four increasingly strict partitioning schemes over the Universal Foundation Set (Table 1). The Cold‐Drug/Warm‐Target partitioning scheme involves drugs being withheld, whereas kinase targets overlap with pretraining; this is the main test condition and is not strictly zero‐shot in terms of the proteins involved. The Scaffold Split condition tests generalization over the chemical structure by partitioning according to Bemis–Murcko scaffolds. Cold‐Target/Warm‐Drug involves proteins that are unseen but drugs which are seen, whereas the Double‐Cold partitioning scheme withholds both. Each result is reported as Mean ± SD across three random seeds.

The ∼11‐point drop in AUROC for scaffold splitting relative to cold‐drug evaluation implies that some fraction of cold‐drug performance reflects molecule‐specific rather than scaffold‐level generalization. Notably, cold‐target generalization (0.844) exceeds scaffold generalization (0.808), consistent with conserved binding pocket structure across the kinome, making protein‐side extrapolation more tractable than scaffold‐level chemical extrapolation.

To mitigate data contamination between the Universal Foundation Set and downstream benchmarks, we performed an exact‐match intersection test on RDKit‐canonicalized SMILES strings. This revealed that 16.2% of Davis drugs and 2.8% of KIBA drugs appeared in the pretraining corpus. All overlapping compounds were removed to create “Strict” benchmark variants consisting entirely of unseen compounds, enabling strict zero‐shot evaluation.

When evaluated on the Strict KIBA benchmark, MVLM achieves an AUROC of 0.930 and an AUPRC of 0.977. These results show that a state‐of‐the‐art performance is maintained even under strict zero‐shot conditions. This confirms that MVLM has learned transferable physicochemical interaction patterns rather than memorizing the training compounds.

As shown in Table 2, conventional deep learning methods like DeepDTA [6] fail to generalize across the structural diversity of the evaluation set, reporting an AUROC of 0.830. More recent transformer‐based and multiview fusion methods such as MolTrans [20] and MDF‐DTA [15] report higher performance at the expense of increasingly complex architectures. MVLM surpasses all baselines with an AUROC of 0.934, demonstrating the robustness of geometric manifold alignment for predicting interactions within heterogeneous chemical libraries.

**TABLE 2:** Cold‐drug holdout evaluation on the Universal Foundation Set. All models were evaluated on completely unseen drug scaffolds spanning 447 human kinases.

Model	Architecture	AUROC ↑
DeepDTA [6]	1D CNN + concatenation	0.8300
MolTrans [20]	Transformer + interaction module	0.9051
MDF‐DTA [15]	Multiview distributed fusion	0.9218
**MVLM (ours)**	**Linear manifold alignment**	**0.9338**

*Note* : Bold indicates the best AUROC.

### 3.2 Comparison Against State‐of‐the‐Art

**Evaluation Protocol and Class Imbalance.** Davis and KIBA were initially developed for continuous affinity regression. However, recent works such as MolTrans [20] and MDF‐DTA [15] report binary classification metrics, and we follow this convention to ensure comparability (Table 3). All baselines share identical active/inactive thresholds (≤100 nM for Davis; KIBA score ≤12.1 for KIBA), identical train/test splits, and identical binary formulation. Baseline metrics are sourced from original publications or the MDF‐DTA and iNGNN comparative analysis where the same protocol was applied. These performance differences reflect representation, pretraining, and interaction design together. Since the 100‐nM activity threshold introduces class imbalance, AUROC is not sufficient; therefore, we also report area under the precision–recall curve (AUPRC). While DeepDTA [6] achieves an AUPRC of 0.743 on the KIBA dataset, MVLM achieves an AUPRC of 0.977, an F1‐Score of 0.906, and a precision of 0.939. This demonstrates the strong performance of MVLM even under class imbalance. The performance of all newly introduced baselines (MolTrans, DrugBAN, ConPLex, MDF‐DTA, and InceptionDTA) was evaluated on identical train/test splits, identical binary formulation. Metrics for DeepDTA and GraphDTA are sourced from their respective publications and the MDF‐DTA comparative evaluation. We note that MVLM additionally benefits from pretraining on 473 923 BindingDB interactions and pretrained ChemBERTa and ESM‐2 encoders, which older baselines do not employ.

**TABLE 3:** State‐of‐the‐art benchmark comparison on Davis and KIBA. Baseline metrics are sourced from published evaluations and recent comparative analyses [15, 21].

Model	Architecture	Year	Davis		KIBA	
			AUROC ↑	AUPRC ↑	AUROC ↑	AUPRC ↑
DeepDTA [6]	1D CNN	2018	—	0.378	—	0.743
GraphDTA [7]	GNN	2020	—	0.316	—	—
MolTrans [20]	Transformer	2021	0.904	0.371	0.898	0.691
DrugBAN [22]	Bilinear attention	2023	0.910	—	0.904	—
ConPLex [19]	PLM + contrastive	2023	0.912	—	0.908	—
MDF‐DTA [15]	Multiview fusion	2024	0.913	—a	0.906	—a
InceptionDTA [23]	Multiscale CNN	2025	0.910	—	0.915	—
iNGNN‐DTI [21]	Nested GNN + PLM	2024	0.931	0.473	0.915	0.753
**MVLM (ours)**	**Linear manifold**	**2026**	**0.928** **±** **0.002**	**0.602** **±** **0.001**	**0.936** **±** **0.004**	**0.977** **±** **0.001**

*Note* : Missing AUPRC values are marked with “—.” Bold indicates the best value in each column.

a MDF‐DTA is a regression model, and its reported AUPRC on the Davis dataset (0.792) is based on continuous pKd predictions. Therefore, in order to ensure consistency in comparing AUPRC scores, all the reported AUPRC values are taken from Sun et al. [21], where binary classifiers are used for predicting the scores.

One of the most competitive recent baselines is iNGNN‐DTI [21], which utilizes three‐dimensional protein structures predicted by AlphaFold2. It processes them with a nested graph neural network along with a cross‐attention module in order to capture substructure‐level drug–target interactions. iNGNN‐DTI also integrates features derived from Chemformer, a large pretrained molecular language model, and ESM for protein representation. On the Davis benchmark, the AUROC and AUPRC values of iNGNN‐DTI are 0.931 ± 0.003 and 0.473 ± 0.017, respectively. MVLM achieves a comparable AUROC of 0.928 ± 0.002 and a substantially higher AUPRC of 0.602 ± 0.001. The difference in the Davis AUROC between MVLM and iNGNN‐DTI (0.003) is within iNGNN‐DTI’s own reported standard deviation, indicating that the two models are statistically indistinguishable on this metric. On the KIBA benchmark, MVLM outperforms iNGNN‐DTI on both metrics (AUROC of 0.936 vs. 0.915 and AUPRC of 0.977 vs. 0.753). iNGNN‐DTI relies on AlphaFold2‐derived protein contact maps as an additional structural input. This is a computationally expensive modality that is unavailable to MVLM, which achieves competitive or superior performance using only frozen sequence embeddings and two learned linear projection matrices.

Two approaches to predicting drug–target interactions have been recently introduced utilizing embeddings from pretrained language models and could be directly compared with MVLM. The first is PMMR [24], which involves frozen embeddings from the PLM and subsequent additional transformer encoder layers with multihead self‐attention and learned attention pooling. The second is MGF‐DTA [25], which involves frozen embeddings from ChemBERTa with Morgan fingerprints combined through gated fusion and ESM‐2 embeddings with k‐mer frequency features through residual fusion, followed by hierarchical attention pooling. Unlike those methods, MVLM uses all of its foundation encoders in the frozen state and only learns two linear projection matrices, where the binding affinity is calculated by a single dot product. To make the comparison fair, all models have been trained and tested on identical splits of the strict KIBA benchmark (seed = 42). PMMR and MGF‐DTA are regression models, and their continuous predictions were transformed into binary classification using the traditional KIBA activity threshold (score <12.1). Table 4 shows the performance of MVLM with an AUROC of 0.930 and an AUPRC of 0.977. It outperforms both other models on all metrics.

**TABLE 4:** Comparison of MVLM versus recently published PLM‐based drug–target interaction methods on the strict KIBA benchmark (seed = 42). All models trained and tested on identical splits. AUROC and AUPRC computed using standard KIBA activity threshold (score < 12.1).

Model	Interaction module	AUROC ↑	AUPRC ↑
PMMR [24]	GCN + transformer	0.815	0.939
MGF‐DTA^a^ [25]	Gated fusion + hierarchical attention	0.896	0.779
**MVLM (ours)**	**Linear dot product**	**0.930**	**0.977**

*Note* : Bold indicates the best value in each column.

a The MGF‐DTA repository does not include a public model implementation. Results reported for a faithful reimplementation of the architecture described in Ni et al. [25].

An alternative line of research treats drug–target affinity prediction as a regression problem for estimating the values of binding constants, such as *K*_d_ or *K*_*i*_. Some recent regression‐based methods are KANPM‐DTA [26] and TSEDTA [27]. In contrast, Gorantla et al. [28] propose a contrastive learning framework in which both chemical and protein encoders undergo end‐to‐end fine‐tuning as opposed to being frozen. Although these approaches have shown strong performance on their respective benchmarks, their evaluation metrics (mean squared error, concordance index) are not directly comparable to those used in this work (AUROC, AUPRC). MVLM is distinct from the above methods in that all foundation encoders are frozen, while only two linear projection matrices are learned, preventing overfitting on small benchmark datasets.

**Statistical Significance of Performance Improvements: McNemar’s Test.** To ensure statistical validity, proxy InceptionDTA [23] was trained and evaluated on exactly the same train/test split as MVLM, enabling paired predictions on identical test examples as required by McNemar’s test. To verify that the performance gain of MVLM over the strongest baseline (InceptionDTA [23]) is statistically significant, we compared decision boundaries optimized for the F1‐Score. MVLM attained an accuracy of 87.7% on the KIBA test set compared to 75.6% for the InceptionDTA. A paired McNemar’s test showed that MVLM correctly classified 3516 interactions that InceptionDTA misclassified, while InceptionDTA correctly classified only 848 interactions misclassified by MVLM. This more than 4‐fold increase is statistically significant (*χ* ^2^ = 1629.9, *p* < 10^−10^), demonstrating that geometric manifold alignment learns a superior decision boundary compared to deep architecture models.

### 3.3 Ablation Study

To understand how each component contributes separately to the performance of MVLM, we carried out an ablation study on the KIBA dataset. We start from the single‐view linear baseline and incrementally add components one at a time. The results are summarized in Table 5.

**TABLE 5:** Ablation study of MVLM on the KIBA dataset where we incrementally add components to a single‐view linear baseline trained from scratch using standard BCE loss. Results are mean ± SD across three random seeds. The statistical significance of the improvements are ascertained through McNemar’s test (*p* < 0.0001, McNemar’s test).

Model configuration	**AUROC** **↑**	ΔAUROC
Baseline (single‐view linear, no pretraining)	0.863 ± 0.011	—
+ Hybrid contrastive loss	0.898 ± 0.005	+ 0.035
+ Multiview drug input	0.916 ± 0.008	+ 0.018
+ Universal pretraining (final MVLM)	0.936 ± 0.004	+ 0.020

We observe in Table 5 that all three components contribute meaningfully to MVLM’s final performance. Adding the InfoNCE contrastive loss results in the largest individual gain (+0.035 AUROC), enforcing geometric selectivity in the shared manifold. Universal pretraining on 473 923 interactions provides the final +0.020 AUROC improvement. This demonstrates that pretraining on large volumes of interaction data enables the model to learn transferable chemical patterns beyond the scope of smaller benchmark datasets.

**Isolating the Geometric Alignment Contribution.** We ask whether performance gains stem from geometric alignment itself or simply from the quality of the foundation model embeddings. To test this, we concatenated ChemBERTa and ESM‐2 features and trained three nonlinear baselines: a deep Multi‐Layer Perceptron (MLP), a Random Forest, and XGBoost (Table 6). On Davis, the deep MLP achieves an AUROC of 0.909, while MVLM achieves 0.928 using only a linear dot‐product interaction. These results demonstrate that geometric alignment, not embedding quality alone, drives the performance gain.

**TABLE 6:** Geometric alignment versus feature concatenation on the Davis dataset. MVLM is compared against nonlinear baselines trained on concatenated ChemBERTa and ESM‐2 features to isolate the contribution of geometric manifold alignment independent of embedding quality.

Approach	Model	**AUROC** **↑**
Feature concatenation	Deep MLP (nonlinear)	0.909
Feature concatenation	Random Forest	0.921
Feature concatenation	XGBoost	0.922
**Geometric alignment**	**MVLM (linear dot product)**	**0.928**

*Note* : Bold indicates the best AUROC.

### 3.4 Discovery Engine Validation

MVLM was applied to a panel of 15 FDA‐approved kinase inhibitors, withholding all target information during the inference phase. Success is defined as retrieval of the drug’s primary clinical target within the top‐5 predictions out of 447 kinases. The results are shown in Table 7. MVLM achieves an MRR of 0.630, Recall@5 of 80.0%, and a median rank of 2 out of 447. Aspirin, included as a negative control, returns no kinase target with confidence above 0.05. Five of the 15 inhibitors are present in BindingDB and may have contributed to pretraining, and a fully held‐out external panel is left to future work.

**TABLE 7:** Discovery engine validation on 15 FDA‐approved kinase inhibitors.

Drug	Indication	Rank	Target	Confidence
Imatinib	CML/GIST	1	**KIT**	>0.98
Dasatinib	CML	4	**PDGFRB**	>0.97
Sorafenib	Renal/Liver cancer	1	**BRAF**	>0.95
Lapatinib	Breast cancer	2	**EGFR**	>0.96
Erlotinib	NSCLC	1	**EGFR**	>0.97
Vemurafenib	Melanoma	2	**BRAF**	>0.96
Ruxolitinib	Myelofibrosis	1	**JAK2**	>0.97
Crizotinib	NSCLC	10	**ALK**	>0.91
Gefitinib	NSCLC	1	**EGFR**	>0.98
Sunitinib	RCC/GIST	1	**KDR**	>0.96
Pazopanib	RCC	2	**KDR**	>0.95
Bosutinib	CML	14	**SRC**	>0.88
Palbociclib	Breast cancer	2	**CDK4**	>0.94
Ribociclib	Breast cancer	36	**CDK4**	>0.79
Axitinib	RCC	1	**KDR**	>0.97
Aspirin	Negative control	—	—	<0.05

*Note* : Known primary targets are shown in bold. Confidence reflects the geometric similarity score $\langle {\hat{z}}_{d},{\hat{z}}_{p}\rangle /\tau$.

The three outliers are biologically interpretable: bosutinib is a promiscuous inhibitor with no single dominant kinase target, crizotinib has MET as a coprimary target alongside ALK, and ribociclib targets CDK4, a cell‐cycle kinase structurally distinct from the tyrosine kinases dominating pretraining data.

The enrichment factor at rank 5 (EF@5) is the ranking‐based measure of retrieval performance in relation to random screening, where:

(6) $$\text{EF@5}=\frac{\text{hits among top 5}/5}{\text{known positives}/447}$$

The average EF@5 achieved by MVLM across the 15 FDA‐approved drugs is 68.1, representing a 1.69‐fold improvement over MolTrans (40.4), as shown in Table 8. Both models share an identical median rank of 2, suggesting that they tend to rank the true target near the top. The differences in Recall@5 and EF@5 arise from MVLM’s stronger geometric separation between correct and incorrect targets within the shared manifold, leading to more consistent rankings across chemically diverse inhibitor scaffolds.

**TABLE 8:** Discovery engine retrieval metrics for MVLM and MolTrans across a panel of 15 FDA‐approved kinase inhibitors screened against 447 human kinases.

Metric	MVLM (ours)	MolTrans
Mean reciprocal rank	**0.630**	0.554
Recall@5	**80.0%**	66.7%
Median rank/447	**2.0**	2.0
Mean EF@5	**68.1**	40.4

*Note* : Bold indicates the better value for each metric.

### 3.5 Quantitative Manifold Interpretability

To evaluate whether the MVLM learned manifold carries biological meaning beyond the Discovery Engine examples, we estimated protein clustering using k‐NN purity with *k* = 5. To verify that MVLM learned additional structure rather than inheriting it directly from ESM‐2 embeddings, we computed k‐NN purity on raw ESM‐2 embeddings as a control (Table 9). MVLM increases global purity from 69.8% to 82.9%, Tyrosine Kinase family purity from 70.5% to 85.3%, and CMGC family purity from 78.2% to 86.5%, confirming that the manifold alignment actively reorganizes the protein embedding space beyond what ESM‐2 encodes alone.

**TABLE 9:** k‐NN purity (*k *= 5) of kinase family clustering in raw ESM‐2 embeddings versus the MVLM‐learned manifold.

Metric	Raw ESM‐2	MVLM (ours)
Global purity	69.8%	**82.9%**
TK family purity	70.5%	**85.3%**
CMGC family purity	78.2%	**86.5%**

*Note* : Bold indicates the higher purity for each metric.

## 4 Discussion

Latent space alignment of foundation models achieves superior performance over complex neural networks trained from scratch. This alignment also recovers a biologically meaningful structure within the kinome without explicit supervision.

**Why Linear Projections Suffice.** ChemBERTa [11] and ESM‐2 [12] already encode rich chemical and evolutionary information. The primary challenge is therefore not learning new representations, but aligning two pretrained embedding spaces into a common geometric framework. High benchmark performance suggests that kinase–ligand interactions are well captured by foundation model embeddings. These embedding spaces are consequently largely linearly alignable.

**Geometric Transparency and Emergence of Phylogenetic Structure.** Since MVLM relies on geometric similarity rather than deep nonlinear layers, interaction scores are directly readable as cosine similarities. Each score reflects the alignment of drug and protein representations in the shared manifold. Kinases within the same evolutionary family cluster more closely than those from different families. This suggests that the learned manifold structure preserves the phylogenetic organization without explicit supervision. This emergent biological structure indicates that MVLM is sensitive to intrinsic properties of the kinome rather than memorizing interaction pairs. We note that geometric transparency achieved through interaction scores as cosine similarities in a shared manifold is distinct from feature‐level interpretability methods such as SHAP. Furthermore, kinase‐family clustering in the shared manifold is a result of learned reorganization rather than direct inheritance of sequence‐level structure, as confirmed by the k‐NN purity control (Table 9).

**Comparison With Deep Learning Architectures.** Most recent DTI architectures use deep convolutional, graph‐based, or transformer models that learn representations and interactions simultaneously. While expressive, these models tend to be task‐specific and parameter‐intensive. Instead, MVLM uses pretrained embeddings and linear projections, minimizing model complexity and the risk of overfitting. The fact that geometric alignment achieves state‐of‐the‐art performance despite its simplicity suggests that representation quality and geometric correspondence outweigh architectural depth for kinase‐focused DTI prediction.

**Computational Efficiency.** The simplicity of MVLM yields direct computational benefits. Training involves only two projection matrices, and inference reduces to a single dot‐product operation. This enables large‐scale virtual screening of compound‐kinase pairs with minimal memory and computational cost as an epoch can be trained in under half a minute in this architecture.

**Limitations and Future Directions.** The linear alignability assumption holds particularly well for kinases, which share conserved structural motifs. Therefore, alignment may not generalize to more heterogeneous protein families. Model performance is also dependent on the quality and coverage of the underlying foundation model embeddings that may not uniformly represent all regions of the chemical and sequence space. The current framework also does not incorporate three‐dimensional structural dynamics or cellular context, both of which can affect the binding behavior in biological systems. Future work should extend this alignment strategy beyond the kinome to broader target classes. The integration of structural data, mutational information, and disease‐specific context warrants investigation.

In the current framework, all layers of ChemBERTa and ESM‐2 contribute equally to the frozen embeddings. Competing approaches either fine‐tune encoders end‐to‐end [28] or add deep interaction modules over fully frozen embeddings [24]. A principled middle ground would selectively adapt only those layers whose features are least transferable to the kinase interaction domain. Future work could apply layer applicability metrics such as those in CactusNets [29] to identify which transformer layers encode the most transferable chemical and evolutionary features, thus enabling more selective and theoretically grounded encoder usage than either full fine‐tuning or uniform freezing.

## 5 Conclusions

We reframe drug–target interaction prediction as a geometric alignment problem rather than a feature‐learning problem, in an approach we call MVLM. Supported by ablation and Discovery Engine validation, MVLM achieves state‐of‐the‐art performance on KIBA and Davis benchmarks, demonstrating that data scale and representation quality outweigh architectural complexity. The learned manifold captures a transferable and biologically meaningful geometry of the kinome. This geometry provides interpretability that is absent in black‐box deep learning approaches. Our work suggests that leveraging the latent knowledge of pretrained foundation models offers a scalable and transparent path toward expanding kinase‐focused drug discovery to the broader druggable proteome. Future work will also investigate layer‐selective transfer learning architectures [29] as a principled alternative to uniform encoder freezing.

## Funding

The authors have nothing to report.

## Conflicts of Interest

The authors declare no conflicts of interest.

## Data Availability

The Universal Foundation Set was constructed from the publicly available BindingDB database (January 2026 release, https://www.bindingdb.org). Model implementation, training scripts, pretrained weights, preprocessing pipelines, and comparison scripts are available at https://github.com/deepdrugai/MVLM‐DT.
